# Pore science and engineering: a new era of porous materials

**DOI:** 10.1093/nsr/nwaf258

**Published:** 2025-06-25

**Authors:** Shen Yu, Li-Hua Chen, Ming-Yuan He, Bao-Lian Su

**Affiliations:** State Key Laboratory of Advanced Technology for Materials Synthesis and Processing, Wuhan University of Technology, China; State Key Laboratory of Advanced Technology for Materials Synthesis and Processing, Wuhan University of Technology, China; Shanghai Key Laboratory of Green Chemistry and Chemical Processes, East China Normal University, China; State Key Laboratory of Advanced Technology for Materials Synthesis and Processing, Wuhan University of Technology, China; Laboratory of Inorganic Materials Chemistry (CMI), University of Namur, Belgium

## Abstract

This perspective introduces a new discipline of pore science and engineering, calling for concerted efforts among researchers to propel porous materials into a new era of theory guide and molecular-level design.

## CLASSIFICATION OF POROUS MATERIALS: FROM POROUS MATERIALS 1.0 TO 2.0

### Porous materials 1.0

**Figure 1.**
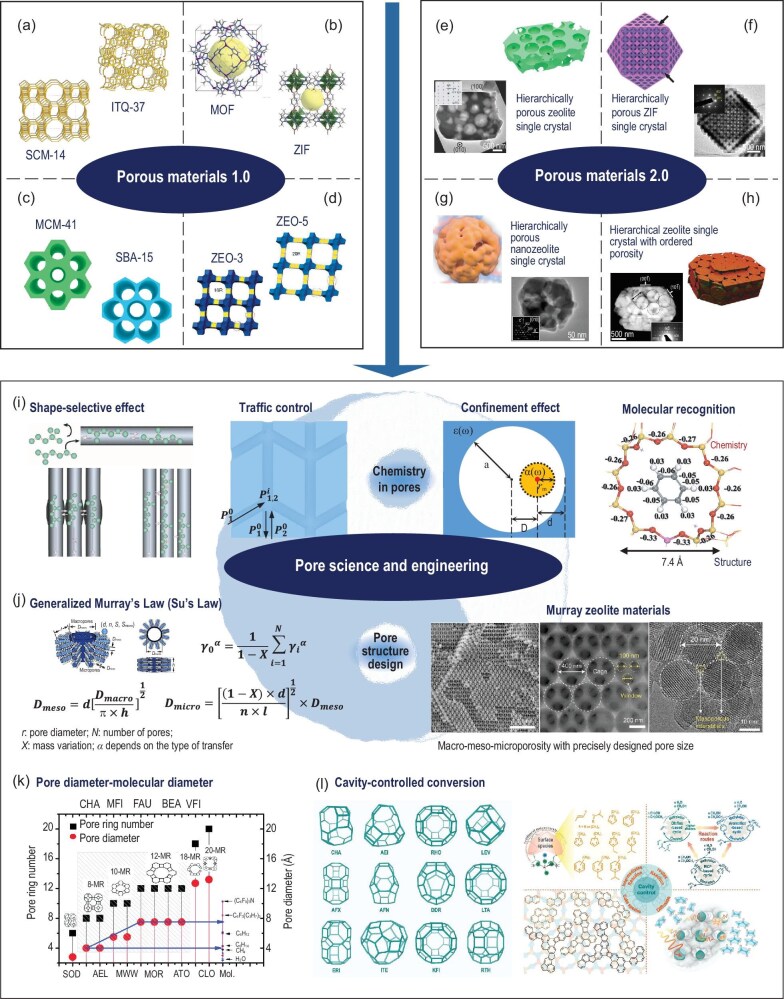
Milestones of porous materials with monomodal pore structures (Porous materials 1.0): (a) new zeolite topology, (b) organic zeolites, (c) mesoporous materials, and (d) extra-large pore zeolites. Schematics of some novel hierarchical porosity inside one single crystals for Porous materials 2.0: (e) hierarchical zeolite, (f) hierarchical ZIF, (g) hierarchical nanozeolite, and (h) hierarchical zeolite with ordered porosity. Adapted with permission from refs [2,4,5,8]. Copyright from International Zeolite Association, (2012, 2019, 2020) American Chemical Society, (2024) Wiley‐VCH GmbH, and (2020) Elsevier B.V. Pore science and engineering: (i) the behavior of guest molecules in pores (chemistry in pores) and (j) pore structure design. Adapted with permission from refs [9,14]. Copyright from (2015) Royal Society of Chemistry and (2023) Oxford University Press. (k, l) Pioneering investigations and understandings of the relationship between pore structure and catalytic performance. Adapted with permission from refs [15,16]. Copyright from (2015) Royal Society of Chemistry and (2023) Oxford University Press.

Porous materials have existed since the birth of our planet, and their utilization in agriculture, building construction, and painting commenced with the advent of civilization. Zeolites, regarded as molecular sieves and the most archetypal porous materials, are a type of microporous material characterized by well-defined crystalline frameworks, uniform and monomodal porosity, molecule-sized windows, and atomically precise chemistry. The successful synthesis of zeolite Y in the laboratory in 1951 (i.e. 200 years after its discovery in nature) and its subsequent utilization as a catalyst in the process of crude oil cracking in 1956 have had a profound impact on petroleum processing and the chemical industry, marking a significant milestone in the field [1]. Since then, a considerable number of zeolites with novel topologies have been synthesized (Fig. 1a and Fig. S1). The chemical composition has undergone significant expansion from the original aluminosilicate to aluminophosphate (ALPO), aluminosilicophosphate (SAPO), and metallosilicophosphate (MSPO), among others. These novel zeolites have found firm applications in catalysis, separation, and ion exchange. The demand for diversity and multifunction of pore architectures in catalysis, gas storage, and separation has led to the development of numerous novel porous materials, including metal-organic frameworks (MOFs, MAFs, ZIFs), polymeric frameworks (PCPs, PAFs), covalent organic frameworks (COFs), zeolite with organic-bridged frameworks (ZOFs), and others (Fig. 1b and Fig. S1) [2]. These microporous materials, which possess controllable pore topologies and inorganic-organic or even pure organic frameworks, have significantly enriched the family of porous materials and remain very active research topics today.

In the early 1980s, there was a growing recognition within the oil refining industry of a significant challenge posed by the increasing heaviness of crude oil. This challenge was compounded by the rapid deactivation of catalysts, a phenomenon that was attributed to molecular diffusion within zeolites, which had narrow pore sizes <1 nm. The development of large-pore molecular sieves emerged as a pressing challenge in the field of porous materials. The initial approach to addressing this issue was to expand the pore size of zeolites, which led to the emergence of a new family of porous materials, highly ordered mesoporous materials, including MCM-41, SBA-15, and CMK-3, based on silica or carbon (Fig. 1c and Fig. S1) [3]. This novel class of porous materials has rapidly evolved into a prominent field of research owing to their facile synthesis and controllable pore structure. However, due to their amorphous nature, these mesoporous materials did not find their industrial application. The research comes back to the synthesis of zeolite-like porous materials. The recent synthesis of extra-large-pore zeolites composed of 16- or 20-membered rings with a pore diameter of 1.0–1.5 nm marked an important step in the field (Fig. 1d and Fig. S1) [4]. All of the aforementioned porous materials possess a shared characteristic in their pore structure, characterized by a uniform pore size at one specific length scale and thus can be labelled as Porous materials 1.0 (see Supplementary Materials).

While the enlargement of the pore size of microporous zeolites has been demonstrated to enhance molecular diffusion within the zeolite crystals, the deactivation of the catalyst due to molecular diffusion persists as a significant challenge in the domain of zeolites. The crystal size of synthetic zeolites utilized in industry ranges from ∼3–8 μm. Due to diffusion limitation, reactants are capable of diffusing only to a depth of 120–150 nm within the confines of zeolite crystals. The utilization efficiency of zeolites is lower than 40% [5]. Consequently, molecular diffusion limitation results in significant waste of raw materials in chemical reaction processes, leading to rapid catalyst deactivation, increased catalyst regeneration frequency, and substantial energy consumption, which significantly hinders the development of efficient and environmentally friendly chemical processes. The solution of the molecular diffusion problem has the potential to contribute to the sustainable development of the chemical industry and to achieve a substantial reduction in carbon emissions, thereby facilitating the realization of a ‘carbon-neutral future’.

### Porous materials 2.0

Hierarchy is ubiquitous in natural systems, manifesting in diverse levels of complexity ranging from simple unicellular organisms to complex organs such as the lung, blood circuit, kidney, trees, and diatoms. Living systems and their hierarchically porous organizations have evolved and optimized the durability of their structures over hundreds of millions of years. They have also developed the capability to adapt to their external environments, to undergo self-healing, to provide protection and mobility to organisms, to help sense the environment, and to perform many other highly complex functions. Hierarchy, therefore, is the consequence of evolution and is the characteristic of life (Hierarchy Law of Life) [6]. Such hierarchically porous structures facilitate optimal mass transport, diffusion, and exchange of substances, as well as the efficient absorption, conversion, and storage of energy. These natural hierarchically pore structures exhibit three characteristics: multi-levels, interconnectivity, and regularity. This makes them an excellent pore structure model for optimized mass transfer and diffusion [6]. The more elegant route to break the diffusion limitation of guest molecules is therefore the introduction of a bio-inspired hierarchical porosity into porous materials. In 2003, a self-formation phenomenon of the hierarchical pore system with very regular and interconnected micro-meso-macroporosity was discovered by Su *et al.* on the basis of the power of chemistry of metal alkoxides and alkyls. To date, more than 200 kinds of porous materials with varying components, structures, and morphologies have been synthesized via this self-formation phenomenon (Fig. S2) [7]. A significant amount of research has also been dedicated to the construction of a hierarchical pore system in one zeolite crystal, thereby endowing porous catalysts with both optimized mass diffusion ability and high structural stability. Macropores can be introduced into zeolite single crystals by the utilization of silica spheres as a silicon source (Fig. 1e and Fig. S2), employing a dissolution-crystallization method [8]. Polymer spheres were used as templates for the synthesis of hierarchically porous ZIF single crystals (Fig. 1f and Fig. S2) [6–8]. Mesoporous nanozeolite ZSM-5 was reported to undergo a transformation in which the interparticle void of zeolite precursors was converted into intraparticle mesopores via an ‘intraparticle ripening’ process (Fig. 1g and Fig. S2) [8]. A more elaborate template-confined crystallization strategy was developed by Su's group to synthesize a hierarchically porous ZSM-5 zeolite single crystal with ordered mesopores (tetrahedral voids) and macropores (octahedral voids) (Fig. 1h and Fig. S2) [8]. In order to optimize the efficiency of porous materials, a considerable number of pioneering works have been carried out to reveal the relationships between pore structure and catalytic performance. Hierarchy factor (HF) was proposed to evaluate the balance between mesopore generation and micropore preservation in diverse synthesis strategies for hierarchical zeolites (Fig. S2) [6–8]. These hierarchically porous systems, which possess two or more pores of varying sizes across different length scales and characteristics of multi-levels, interconnectivity, and regularity, are designated as Porous materials 2.0 (see Supplementary Materials). The application of hierarchically porous materials has become increasingly prevalent in industrial processes, signifying a growing field of significant interest for both the academic and industrial research communities [6–8].

## PORE SCIENCE AND ENGINEERING

In order to achieve further rational and quantitative design for the synthesis of hierarchically porous materials, it is imperative to develop a theory of pore design for advancing the field of porous materials. It will allow for the maximized utilization of raw materials, minimized energy consumption, and the rational and quantitative design of chemical processes. Firstly, the behavior of guest molecules in the pores, or pore chemistry, is paramount for the design and engineering of pores. Pore chemistry encompasses a number of key effects (Fig. 1i and Fig. S3), including shape-selective effect [9], traffic control [10], confinement effect [11], and molecular recognition effect [12]. These effects have a significant impact on a range of processes, including diffusion, reaction, product formation, and coke deposition. In 1960, Weisz and Frilette demonstrated that diffusion into and out of zeolite pores was possible only for molecules that match the size and shape of the pores [13]. This shape-selective effect was further interpreted as reactant selectivity, transition state selectivity, and product selectivity (Fig. 1i and Fig. S3) [9]. Subsequently, Derouane and Gabelica put forth the concept of ‘molecular traffic control’ to generalize the observation that reactant molecules preferentially tend to enter into one channel system while products diffuse out by the other pore system. This intriguing phenomenon, as demonstrated in Fig. 1i and Fig. S3, effectively prevents occurrence of the counter-diffusion limitation in the methanol to hydrocarbon (MTH) reaction [10]. The confinement imposed by energy fields is often neglected. To illustrate the diffusion behavior of molecules with the same van der Waals radius of both the pore and chain molecules along the channel wall, Derouane *et al.* employed the concepts of ‘floating molecule’ and ‘creep diffusion’, respectively (Fig. 1i and Fig. S3) [11]. In 1992, Barthomeuf and Su discovered a selective adsorption phenomenon that depends on both structural properties (e.g. pore diameter) and chemical properties (e.g. electron densities of adsorbate and zeolite) as the phenomenon of recognition between enzymes and substrates in biology (Fig. 1i and Fig. S3) [12]. This fundamental phenomenon, known as ‘molecular recognition’, plays a pivotal role in the modulation of product selectivity for the alkylation reaction of aromatics. The rational design of the framework composition and proper modification of the pore surface can optimize the behavior of guest molecules in pores, thereby achieving controllable and desirable reaction pathways. However, this process depends significantly on the development of quantitative principles for energy fields, electronic clouds, electronegativity, wettability, and others in porous frameworks (see Supplementary Materials). Pore chemistry constitutes an indispensable part of pore science and engineering.

In addition to the field of pore chemistry, the rational design and engineering of pore structures, for instance, the optimal pore size distribution for macropores, mesopores, and micropores, was still in vague, which restrains the optimization of pore structure and thus the maximization of catalytic efficiency. Su *et al.* revisited ‘Murray's Law’ by taking mass variation during chemical reactions and surface effect of molecular diffusion into consideration and established the ‘Generalized Murray's Law’ (also called ‘Su's Law’) for designing hierarchically porous materials (Fig. 1j and Fig. S2) [6,8,14]. On the basis of such law, a hierarchically macro-meso-microporous ZSM-5 zeolite Murray material with pore distribution obeying the generalized Murray's Law was prepared (Fig. 1j and Fig. S2) [14]. The diffusion rate and cracking performance of the Murray ZSM-5 zeolite were enhanced by a factor of ∼1 order of magnitude. In order to achieve the objective of optimal fitness between hierarchical structure and diffusion ability, it is imperative in Su's Law to take into account more additional parameters (descriptors) that significantly influence mass transport and diffusion in future work, including the number of hierarchy levels, number and angle of branches, porosity characteristics such as pore volume, length, stability, and flexibility, as well as chemical composition, pressure, and temperature for a given chemical reaction. The establishment of a more accurate mathematical expression from Su's Law with different descriptors of porous materials will be an important research direction in the future. Pore structure design, synthesis, and engineering is another important part of pore science and engineering.

Last, but not least, for a specific chemical reaction, the pore structure, chemical composition, active site concentration, and location are the focal points of pore science and engineering research. Xie and Tang *et al.* provided a comprehensive overview of the correlation between molecular diameter, pore diameter, and pore ring number across various zeolites, with a focus on their application in industrial oil refining and petrochemical processes (Fig. 1k) [15]. Liu's group has made significant contributions to the field of zeolite-catalyzed methanol-to-olefins (MTO) reaction through their pioneering works. They have developed a methodology for selecting zeolite with a specific cavity size to regulate reaction intermediates, reaction routes, coke species, and diffusion (Fig. 1l) [16]. The aforementioned principles have contributed to the transformation of a research model from ‘trial-testing-modification-retesting’ to a more reactive approach of ‘reaction-demanded synthesis’ in the design, synthesis, and engineering of porous materials, which will contribute to the expansion of the connotation of pore science and engineering.

From the law of hierarchy, a new discipline of ‘Pore Science and Engineering’ can be created on the basis of the immense amount of literature, accelerating the development of porous materials. Artificial intelligence can facilitate the construction of this new discipline by analysis and assembly of largely dispersed literature. Future works are encouraged to focus on the understanding of molecule behavior in pores, the development of the theory of design and construction (size, geometry, length, *etc*.) and the establishment of mathematical expression with different descriptors of a porous material. Porous materials are envisioned to enter a new era of theory to guide molecule-level design toward an optimized structure and advanced functions for industrial processes with maximized efficiency and minimized energy consumption. Pore Science and Engineering, as a new discipline is still in its infancy, and its development requires a collaborative effort among researchers in the broader field of porous materials research. This emerging discipline necessitates the active involvement of individuals, particularly young scholars, and is poised to become a significant source of new ideas and innovation, thereby contributing to the sustainable development of society and the achievement of a ‘carbon-neutral future’.

## Supplementary Material

nwaf258_Supplemental_File

## References

[1] Day GS, Drake HF, Zhou HC et al. Commun Chem 2021; 4: 114. 10.1038/s42004-021-00549-4 36697550 PMC9814869

[2] Zhang JP, Zhang YB, Lin JB et al. Chem Rev 2012; 112: 1001–33. 10.1021/cr200139g 21939178

[3] Li W, Liu J, Zhao D. Nat Rev Mater 2016; 1: 16023. 10.1038/natrevmats.2016.23

[4] Yu H, Villaescusa LA, Gao ZR et al. Angew Chem Int Ed 2024; 63: e202412170. 10.1002/anie.202412170 39142293

[5] Sun MH, Zhou J, Hu ZY et al. Matter 2020; 3: 1226–45. 10.1016/j.matt.2020.07.016

[6] Chen LH, Li Y, Su BL. Natl Sci Rev 2020; 7: 1626–30. 10.1093/nsr/nwaa251 34691495 PMC8290953

[7] Sun MH, Huang SZ, Chen LH et al. Chem Soc Rev 2016; 45: 3479–563. 10.1039/C6CS00135A 27255561

[8] Chen LH, Sun MH, Wang Z et al. Chem Rev 2020; 120: 11194–294. 10.1021/acs.chemrev.0c00016 32915551

[9] Wei Y, Parmentier TE, de Jong KP et al. Chem Soc Rev 2015; 44: 7234–61. 10.1039/C5CS00155B 26007224

[10] Derouane EG, Gabelica Z. J Catal 1980; 65: 486–9. 10.1016/0021-9517(80)90328-0

[11] Derouane EG, André JM, Lucas AA. Chem Phys Lett 1987; 137: 336–40. 10.1016/0009-2614(87)80895-3

[12] Su BL . J Chem Soc Faraday Trans 1997; 93: 1449–57. 10.1039/a607015i

[13] Weisz PB . Pure Appl Chem 1980; 52: 2091–103. 10.1351/pac198052092091

[14] Mintova S . Natl Sci Rev 2023; 10: nwad155. 10.1093/nsr/nwad155 37377848 PMC10292680

[15] Shi J, Wang Y, Yang W et al. Chem Soc Rev 2015; 44: 8877–903. 10.1039/C5CS00626K 26567526

[16] Zhang W, Lin S, Wei Y et al. Natl Sci Rev 2023; 10: nwad120. 10.1093/nsr/nwad120 37565191 PMC10411685

