# Supplementary material for: Pore science and engineering: a new era of porous materials
Source: Natl Sci Rev. 2025 Jun 25;12(8):nwaf258. doi: 10.1093/nsr/nwaf258 (PMC12343094; doi:10.1093/nsr/nwaf258)
Supplement: nwaf258_Supplemental_File [file nwaf258_supplemental_file.pdf]

Supplementary Materials

Pore science and engineering: a new era of porous materials

Shen Yu[1], Li-Hua Chen[1*], Ming-Yuan He[2,*] and Bao-Lian Su[1,3*]

[1]State Key Laboratory of Advanced Technology for Materials Synthesis and Processing, Wuhan University of Technology, Wuhan 430070, China;

[2]Shanghai Key Laboratory of Green Chemistry and Chemical Processes, East China Normal University, Shanghai 200062, China

[3]Laboratory of Inorganic Materials Chemistry (CMI), University of Namur, Namur B-5000, Belgium

E-mail: chenlihua@whut.edu.cn; hemingyuan@126.com; bao-lian.su@unamur.be

Porous materials like natural zeolites of sedimentary origin and charcoal exist since the birth of our earth and their utilization by mankind in agriculture, constructions and paintings began with our civilization[1]. Writing ink, a mixture of porous charcoal suspended in gum slurry, is the first example of the utilization of man-made porous materials recorded around 2500 BC in ancient Egypt and China, on the basis of the statement of 'Encyclopedia Britannica'[2]. As far back as 4000 years ago, ink also appeared on Chinese pottery. Charcoal is a black porous substance made at that time by burning wood slowly in an oven with little air. Other than used as a preservative by ancient Egypt and China, charcoal was made by prehistoric people to remove impurities from smelting minerals to make bronze and to filter air. Reports on the use of charcoal in medical practices for indigestion can be dating back to circa 1500 BC. Around 400 BC, ancient Indians and Phoenicians began to use it to purify water[1]. Today we know that charcoal is amorphous carbon with a very large distribution of pores and a disordered structure. All the above properties of charcoal come from these pores.

Different from amorphous characteristics of charcoal, natural zeolites, a family of crystalline aluminosilicates with well-organized frameworks and monomodal pore size structure (**Fig. S1**) were discovered by Fredrick Cronsted, a Swedish mineralogist, during the collection of minerals in Lappmark copper mine[3]. He baptized the new mineral with the name 'zeolite', because of the characteristic intumescence of the mineral observed by him during blowpipe tests on the crystals. The term 'zeolite' is derived from two Greek roots 'zeo' to boil and 'lithos' to stone, respectively. Zeolites have found firm applications starting from the fifties of the last century owing to the pioneering works of Barrer who discovered a variety of properties of zeolites, such as physical adsorption, cation exchange, reactivity with alkalis, propensity to thermal expansion and insulation, and he also synthesized the first artificial zeolites[4]. Milton *et al.* produced zeolites from soluble silicon and aluminum precursors and used synthetic zeolites as catalysts because of their strong adsorptive and acid properties[5]. By 1956, Zeolite Y was used as a hydrocarbon isomerization catalyst (**Fig. S1**)[5]. Since then, synthetic zeolites have revolutionized the refinement of crude oil and the petrochemical industry as a whole by vastly improving the efficiencies of the existing processes and their application has been extended to separation and environmental remediation processes and health-care fields. During the past 20 years, plenty of zeolites with new topology have been synthesized, such as JU series by Yu's team, ECNU series by Wu's group, ITQ series invented by Corma's group, and SCM series developed by Yang and Sun (**Figs. 1a** and **S1**). Their well-defined crystalline frameworks, uniform and monomodal porosity, molecule-sized windows and atomically precise chemistry offer unprecedented properties, such as shape

selectivity[6] and molecular recognition[7], which results in a general explosion of zeolite research.

For commodity chemical industry, zeolites present two important drawbacks. Firstly, the very simple aluminosilicate chemical compositions limit zeolites only for acid-basic reactions and shape selective separation. Secondly, their pore size less than 1 nm results in the diffusion problem of bulky molecules. For the first drawback, one of important milestones in the zeolite science is the synthesis of ALPO (aluminophosphates) and SAPO (silicoaluminophosphates) molecular sieves which largely extends the chemical compositions of zeolite related materials made by Flanigen (**Fig. S1**)[8,9]. Scientific interest and industrial need in porous materials have been the driving force for the exceptional growth over the past few decades with the development of modern frameworks. Today, a large series of metal and non-metal ions (B, Ti, Ga, Ge, Sn, *etc.*) can be incorporated zeolite frameworks and more than 250 kinds of zeolites and related materials with different topologies have been synthesized (data from the International Zeolite Association). Zeolites go to organic. The inorganic zeolitic materials meet often organic molecules for catalysis and separation. The low organo-affinity of zeolitic materials and the art of the construction of new frameworks call for organo-inorganic hybrid porous materials. From the beginning of the 1990's, we have witnessed the appearance of a large series of metal-organic frameworks (MOFs, MAFs, ZIFs, *etc.*)[10–12], polymeric frameworks (PCPs, PAFs, *etc.*)[13–15], covalent organic frameworks (COFs, *etc.*)[16], zeolite with organic frameworks (ZOFs)[17], *etc.*, enriching significantly the family of porous materials (**Figs. S1** and **1b**) and remaining very hot research topics today. It is possible that these new porous materials find their victory as zeolites do in other industrial fields other than traditional catalysis, separation and dehydration.

The diffusion and mass transfer of reactive molecules in micropores of zeolitic catalysts significantly affect the whole catalytic process, and the inefficient diffusion and mass transfer directly lead to low catalytic efficiency, poor selectivity and quick catalyst deactivation by coke deposition. To solve the diffusion problem of bulky molecules, there exist two routes. The first one is to expand the pore size of the above-mentioned porous materials. The fact that the size of molecules in crude oils becoming bigger and bigger accelerates this kind of research, which leads to the birth of a new family of porous materials, highly ordered mesoporous materials such as MCM-41[18], SBA-15[19]and CMK-3[20], based on silica or carbon (**Figs. S1** and **1c**). This new family of porous materials attract scientists from different fields due to their easy and well reproducible synthesis and their facile and controllable structure and pore size and quickly became a rapidly evolving field of the important interest.

Recently, Yu's group reported the successful synthesis of extra-large-pore zeolites with 16- or 20-membered rings with a pore diameter of 1.0–1.5 nm (**Figs. 1d** and **S1**)[21–23]. It is worthy to note that these zeolites possess high structural stability for adsorption and catalysis. All the above-mentioned porous materials, in spite of their long evolution, have a common character of the monomodal pore structure and constitute the first generation of porous materials (Porous Materials 1.0 as illustrated in **Fig. S1**).

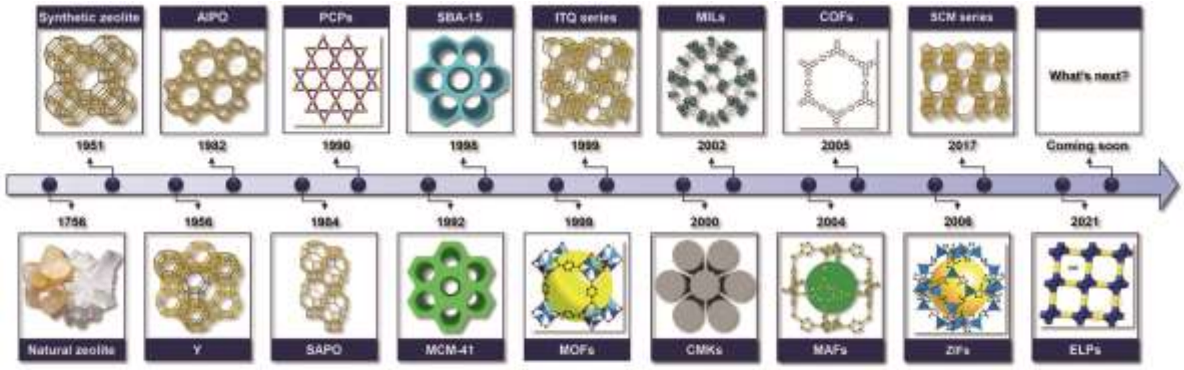

Figure S1. The history of Porous Materials 1.0 with all the pores at same single length scale. Adapted with permission from references[11,12,14,24–26]. Copyright from International Zeolite Association, (2006) National Academy of Sciences of the USA, (2009, 2012, 2021, 2021) American Chemical Society, and (2010) Royal Society of Chemistry.

After millions of years of evolution, natural systems are not only designed and optimized for durability but also possess the capability to adapt to their external environment, to undergo self-repair, and to perform many highly complex functions[27,28]. To achieve mass transfer and exchange with extremely high efficiency while minimum energy consumption, many classes of organisms have evolved to possess hierarchically porous networks, in which the pore sizes regularly decrease (and branch) across multiple scales and finally terminate in size-invariant units, such as those seen in plant stems, leaf veins, vascular and respiratory systems[28]. These hierarchical pore structures with optimized mass transfer and diffusion ability exhibit three characteristics: multi-levels, interconnectivity, and regularity[29–35]. The second and more elegant route to solve the problem of inefficient diffusion of guest molecules in micropores of zeolitic catalysts is bio-inspired by introducing a hierarchical porosity with interconnectivity, defined pore sizes and regularity of different pores. The first artificial hierarchical pore system was originated from a self-formation phenomenon discovered by Su *et al.* in 2003 (**Fig. S2**)[36]. When dropped into water, metal alkoxides was found to be able to transform into hierarchical porous metal oxide through a controllable hydrolysis and condensation chemical processes, during which a large number of alcohol

molecules and water molecules release rapidly and resulted in hierarchically macro-mesoporous structures[36]. This self-formation phenomenon was widely utilized for the fabrication of single metal oxide, binary or ternary metal oxides, and carbon-based materials with dual or triple porosities. So far, more than 200 kinds of porous materials with different components, structures, and morphologies have been synthesized by utilizing self-formation phenomenon[37–40]. These hierarchically porous materials not only exhibited highly improved catalytic efficiencies but also were used as precursors to fabricate hierarchically porous zeolite materials, such as TS-1, Beta, ZSM-5, and so on, exhibiting excellent shape-selective catalytic performance (**Fig. S2**)[37,41]. Since the proposition of 'hierarchically porous materials', considerable effort has been devoted to construct hierarchical pore system in one zeolite single crystals to endow zeolitic catalysts with efficient mass diffusion ability and high stability, aiming to satisfy the harsh reaction environment in industry. Schwieger *et al.* reported that macropores can be introduced into zeolite single crystals by the utilization of silica spheres as a silicon source, employing a dissolution-crystallisation method (**Fig. 1e**)[42]. Chen *et al.* used polymer spheres as templates and the crystallization of ZIF crystals were strictly confined in the voids of close-packed polymer spheres, which finally produced hierarchically macro-microporous ZIF-8 single crystals (**Fig. 1f**)[43]. Yu's group introduced mesopores into nanozeolite ZSM-5 by transforming the interparticle void of zeolite precursors into intraparticle mesopores via an 'intraparticle ripening' process (**Fig. 1g**)[44]. Su's groups used a 3D macro-mesoporous carbon network as hard template and the crystallization of zeolite was strictly confined in the voids of carbon templates. The resultant zeolite single crystal possessed mesopores (tetrahedral voids from a model of face-centered cubic (FCC)-packed spheres) and macropores (octahedral voids from a model of FCC-packed spheres), in addition to its intrinsic micropores (**Fig. 1h**)[45]. The as-obtained hierarchical ZSM-5 zeolite showed a relative diffusion rate 10 and 7 folds higher than that of micro-sized ZSM-5 and nanosized ZSM-5, respectively. The catalytic efficiencies and lifetimes of methanol-to-olefins and catalytic cracking reactions were significantly promoted due to the highly improved diffusion rate and high structural stability endowed by the single crystal structure. This synthetic method was successfully extended for the synthesis of diverse zeolite topologies, such as Beta, TS-1, and SAPO, targeting at different reaction systems[46]. Zhao's group reported a dynamic micelles-templated method to prepare hierarchically porous structure with gradient pore size distribution (**Fig. S2**)[47]. This hierarchical gradient structure enabled capillary-directed fast mass transfer from the solutions to inner active sites and achieved significantly improved catalytic yield and stability on the esterification of long-

chain carboxylic palmitic acids. It was demonstrated that the pore size of each level of hierarchical macro-meso-microporosity in the above-mentioned hard-template method can be precisely tuned. Nevertheless, the optimal pore size distribution for macropores, mesopores, and micropores was still in vague, which severely restrains the optimization of pore structure thus the maximization of catalytic efficiency. In order to explore a design principle for hierarchically porous materials with maximized efficiency, Pérez-Ramírez *et al.* proposed a concept of hierarchy factor as a function of relative mesoporous surface area (S_{meso}/S_{BET}) and relative microporous volume (V_{micro}/V_{pore})[48]. The maximization of the hierarchy factor (HF) signifies the enhancement of the mesopore surface area without significant reduction in micropore volume (**Fig. S2**). This can be accomplished through a desilication process involving NaOH. HF enables the quantitative comparison and correlation of various materials obtained by different synthetic methodologies. However, HF only provides a preliminary principle for selecting a synthesis method for hierarchically porous materials.

Mastering the law of pore size distribution is of great significance for establishing the theory of pore design, synthesizing hierarchically porous materials with high efficiencies, improving the efficiency of chemical reaction processes, maximizing the utilization rate of raw materials, and minimizing energy consumption. In 1926, Murray proposed 'Murray's Law' to describe a principle that quantitatively states the cost of oxygen transport system in human to be a minimum, which shed enlightenment on optimizing individual pores in hierarchical structure for mass transport and diffusion[49]. In 2017, Su *et al.* revisited 'Murray's Law' by taking mass variation during chemical reactions into consideration and obtained 'Generalized Murray's Law' (also called 'Su's Law') that can be applied for designing hierarchically porous materials (**Fig. S2**)[28]. The as-obtained porous materials whose pore sizes are multiscale and are designed with pore diameter ratios obeying the generalized Murray's Law are termed as Murray materials. The first synthetic Murray material, hierarchically macro-meso-microporous ZnO, showed excellent mass transport and diffusion ability and performance in liquid, gas, and solid systems[28]. On the basis of Generalized Murray's Law, a Murray zeolite material, hierarchically macro-meso-microporous ZSM-5 zeolite inverse opal structure, was prepared (**Fig. S2**)[50]. The 1,3,5-trimethyl benzene diffusion rate and 1,3,5-triisopropyl benzene cracking performance of the obtained Murray ZSM-5 zeolite were promoted by almost an order of magnitude, compared to industrial ZSM-5 zeolite. Recently, Hasan *et al.* developed a universal Murray's Law that can be applicable to a wide range of hierarchical structures with diverse pore shapes (**Fig. S2**)[51]. They constructed hierarchical planar and tubular graphene oxide aerogels (GOA) structures to validate the proposed law

and significant improvement was achieved with gas sensor based on the GOA whose pores were optimized by universal Murray's Law. For the goal of perfect fitness between hierarchical structure and diffusion ability, it is imperative in the Su's Law to take into account more additional parameters (descriptors) that significantly influence mass transport and diffusion in future work, including the number of hierarchy levels, then number and angle of branches, the porosity characteristics such as pore volume, length, stability, the chemical composition, pressure, and temperature for a given chemical reaction. The establishment of more accurate mathematical expression from Su's Law with different descriptors of porous materials will be an important research direction in future. The flexibility of porous system is also a very important factor while it is often neglected. For example, it has been demonstrated that framework flexibility generally accelerates the diffusion of tightly fitting molecules. Corma *et al.* found that the zeolite framework can accommodate molecules that are nearly 1 Å larger than the nominal crystallographic pore aperture size[52]. In a recent study, Wei's group reported that the opening pores of the ZSM-5 framework exhibited a maximum aspect change of 15% for the accommodation of benzene molecules. The structural flexibility was achieved by the soft Si-O-Si hinges between rigid tetrahedral SiO_4 units[53].

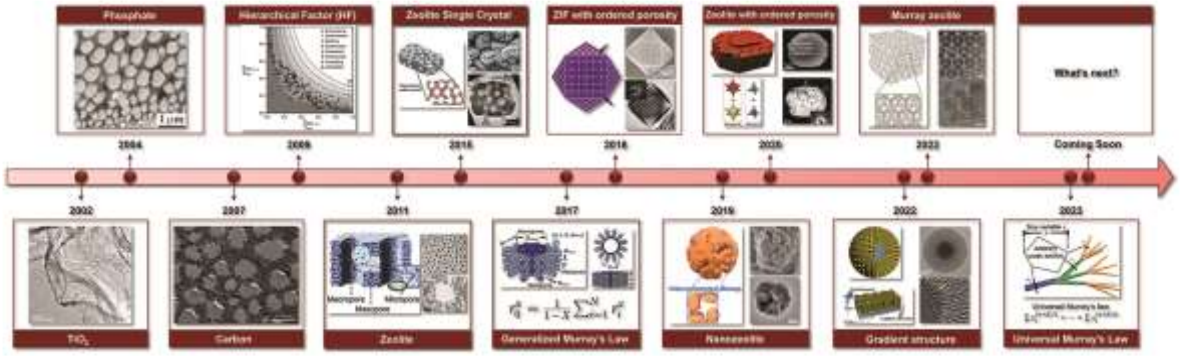

Figure S2. The history of Porous Materials 2.0 with multi-sized pores at different length scales. Adapted with permission from references[28,38–42,44,45,47,48,50,51,54]. Copyright from (2017, 2024) Springer Nature, (2009, 2011, 2015) Wiley‑VCH GmbH, (2022) Oxford University Press, (2007, 2019, 2019, 2022) American Chemical Society, (2020) Elsevier Inc., and (2002, 2004) Royal Society of Chemistry.

Pore chemistry, that is behavior of guest molecules in pores, is also extremely important for pore design and engineering. Pore chemistry (**Figure S3**) includes four distinct effects: shape selective effect[6], traffic control[55], confinement effect[56], and molecular recognition effect[7]. In 1960, Weisz and Frilette found that when zeolites were used as catalysts, the

reaction was mainly carried out in the crystal framework rather than on the surface, and only those molecules that matched the size and shape of the zeolite pores could diffuse into and out of the pores and become reactants and products[57]. This shape selective effect was further interpreted as the diffusion control of reaction direction imposed by special geometric configuration of zeolite, embodying to be reactant selectivity, transition state selectivity, and product selectivity (**Figure S3a**)[6]. The depth understanding of shape selectivity contributed to the thrive development of shape selective catalysis, including catalytic cracking, isomerization, and alkylation reactions. Later, Derouane and Gabelica proposed ‘molecular traffic control’ to generalize the observation that reactant molecules preferentially enter into one channel system while product diffuse out by the other pore system. In a methanol to hydrocarbon (MTH) reaction, reactant molecules diffused into zeolite via nearly-circular sinusoidal channels while products of isoaliphatics, methyl-aliphatics, and monocyclic aromatics desorbed through the linear and elliptical channels, thus preventing the occurrence of counter-diffusion limitation (**Figure S3b**)[55]. Besides the well-known steric confinement effect of pore structure, the confinement imposed by energy field also has an extremely important influence on the diffusion, reactions, products, and carbon formation of molecules but is often neglected. Derouane and André constructed van der Waals model describing the molecule-surface interaction in pores and proposed ‘floating molecule’ and ‘creep diffusion’ to vividly describe the diffusion behavior of molecules with the same van de Waals radius as the pore or molecules (chain molecules) stick to the pore wall, respectively (**Figure S3c**)[56]. In 1992, a selective adsorption phenomenon depending on both structural properties and chemical properties (such as electron densities of adsorbate and zeolite) was discovered by Barthomeuf and Su, named as ‘molecular recognition’[7,58–62]. For example, only when the electron interaction and window morphology of zeolite framework match with benzene molecules, can benzene molecules be selectivity absorbed on 12R window (**Figure S3d**)[7,60,61]. This selective adsorption behavior determined by both chemical and structural properties is similar to an enzyme-substrate system; zeolites can thus be referred to as solid enzymes or zeo-enzymes[60]. This ‘molecular recognition’ phenomenon can be tuned by structural and chemical modifications, such as by introducing a co-adsorbate (HCl, NH_3 or CH_3NH_2)[60], which plays a decisive role in the modulation of product selectivity, such as benzene ring alkylation product or side chain alkylation product in alkylation of toluene[63]. Pore wettability also plays a pivotal role in heterogeneous catalysis. There are also some structural properties that can influence the behavior of molecules. For example, the surface of zeolite can undergo modification to manifest a suite of meticulously tailored wettability

properties. Xiao's group developed a novel method for accelerating the transportation of water products in syngas conversion. They achieved this by combining hydrophobic poly(divinylbenzene) with cobalt-manganese carbide, thereby creating a local environment conducive to rapid water transportation[64]. Wettability constitutes a pivotal parameter within the domain of pore chemistry, exerting a substantial influence on the advancement of pore science and engineering.

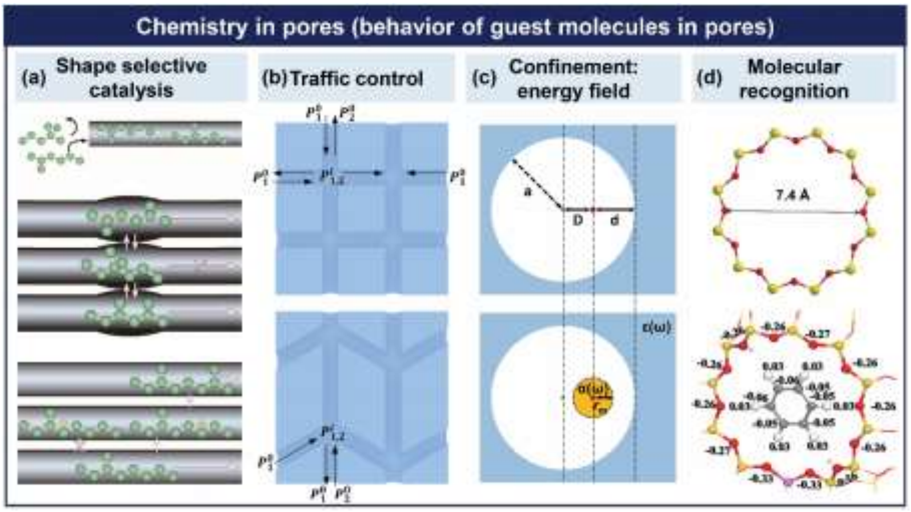

Figure S3. Chemistry in pores: (a) shape selective effect, (b) traffic control effect, (c) Confinement effect by energy field, and (d) molecular recognition effect. Adapted with permission from references[65]. Copyright from (2015) Royal Society of Chemistry.

References

1. Day GS, Drake HF, Zhou HC *et al.* Evolution of porous materials from ancient remedies to modern frameworks. *Commun Chem* 2021; **4**: 114.
2. Cohen N. The new encyclopedia britannica. *Libr J* 2007; **132**: 168.
3. Colella C, Gualtieri AF. Cronstedt's zeolite. *Microporous Mesoporous Mater* 2007; **105**: 213–21.
4. Rees LVC. Richard Maling Barrer. 16 June 1910–12 September 1996. *Biogr Mem Fellows R Soc* 1998; **44**: 37–49.
5. Milton RM. Molecular sieve science and technology. *Zeolite Synthesis*. Vol 398. American Chemical Society, 1989, 1.
6. Smit B, Maesen TLM. Towards a molecular understanding of shape selectivity. *Nature* 2008; **451**: 671–8.
7. Su BL. Molecular recognition effect in benzene adsorption on the 12R window zeolites. *J Chem Soc Faraday Trans* 1997; **93**: 1449–57.
8. Wilson ST, Lok BM, Messina CA *et al.* Aluminophosphate molecular sieves: a new class of microporous crystalline inorganic solids. *J Am Chem Soc* 1982; **104**: 1146–7.
9. Lok BM, Messina CA, Patton RL *et al.* Silicoaluminophosphate molecular sieves: another new class of microporous crystalline inorganic solids. *J Am Chem Soc* 1984; **106**: 6092–3.
10. Li H, Eddaoudi M, O'Keeffe M *et al.* Design and synthesis of an exceptionally stable and highly porous metal-organic framework. *Nature* 1999; **402**: 276–9.
11. Zhang JP, Zhang YB, Lin JB *et al.* Metal azolate frameworks: from crystal engineering to functional materials. *Chem Rev* 2012; **112**: 1001–33.
12. Park KS, Ni Z, Côté AP *et al.* Exceptional chemical and thermal stability of zeolitic imidazolate frameworks. *Proc Natl Acad Sci* 2006; **103**: 10186–91.
13. Ben T, Ren H, Ma SQ *et al.* Targeted synthesis of a porous aromatic framework with high stability and exceptionally high surface area. *Angew Chem Int Ed* 2009; **48**: 9457–60.
14. Janiak C, Vieth JK. MOFs, MILs and more: concepts, properties and applications for porous coordination networks (PCNs). *New J Chem* 2010; **34**: 2366–88.
15. Yaghi OM, Li G, Li H. Selective binding and removal of guests in a microporous metal–organic framework. *Nature* 1995; **378**: 703–6.
16. Côté AP, Benin AI, Ockwig NW *et al.* Porous, crystalline, covalent organic frameworks. *Science* 2005; **310**: 1166–70.
17. Liu Y, Chen P, Wang Y *et al.* Design and synthesis of a zeolitic organic framework. *Angew Chem Int Ed* 2022; **61**: e202203584.
18. Kresge CT, Leonowicz ME, Roth WJ *et al.* Ordered mesoporous molecular sieves synthesized by a liquid-crystal template mechanism. *Nature* 1992; **359**: 710–2.
19. Zhao D, Feng J, Huo Q *et al.* Triblock copolymer syntheses of mesoporous silica with periodic 50 to 300 angstrom pores. *Science* 1998; **279**: 548–52.
20. Jun S, Joo SH, Ryoo R *et al.* Synthesis of new, nanoporous carbon with hexagonally ordered mesostructure. *J Am Chem Soc* 2000; **122**: 10712–3.
21. Pérez-Ramírez J, Verboekend D, Bonilla A *et al.* Zeolite catalysts with tunable hierarchy factor by pore-growth moderators. *Adv Funct Mater* 2009; **19**: 3972–9.
22. Gao ZR, Yu H, Chen FJ *et al.* Interchain-expanded extra-large-pore zeolites. *Nature* 2024; **628**: 99–103.
23. Li J, Gao ZR, Lin QF *et al.* A 3D extra-large-pore zeolite enabled by 1D-to-3D topotactic condensation of a chain silicate. *Science* 2023; **379**: 283–7.
24. Furukawa H, Yaghi OM. Storage of hydrogen, methane, and carbon dioxide in highly

porous covalent organic frameworks for clean energy applications. *J Am Chem Soc* 2009; **131**: 8875–83.
25. Chakraborty G, Park IH, Medishetty R *et al.* Two-dimensional metal-organic framework materials: synthesis, structures, properties and applications. *Chem Rev* 2021; **121**: 3751–891.
26. Cai G, Yan P, Zhang L *et al.* Metal–organic framework-based hierarchically porous materials: synthesis and applications. *Chem Rev* 2021; **121**: 12278–326.
27. Peng Z, Chen LH, Sun MH *et al.* A hierarchical zeolitic Murray material with a mass transfer advantage promotes catalytic efficiency improvement. *Inorg Chem Front* 2018; **5**: 2829–35.
28. Zheng XF, Shen GF, Wang C *et al.* Bio-inspired Murray materials for mass transfer and activity. *Nat Commun* 2017; **8**: 14921.
29. Wu L, Li Y, Fu ZY *et al.* Hierarchically structured porous materials: synthesis strategies and applications in energy storage. *Natl Sci Rev* 2020; **7**: 1667–701.
30. Chen LH, Li XY, Rooke JC *et al.* Hierarchically structured zeolites: synthesis, mass transport properties and applications. *J Mater Chem* 2012; **22**: 17381–403.
31. Sun M, Chen C, Chen L *et al.* Hierarchically porous materials: synthesis strategies and emerging applications. *Front Chem Sci Eng* 2016; **10**: 301–47.
32. Sun MH, Huang SZ, Chen LH *et al.* Applications of hierarchically structured porous materials from energy storage and conversion, catalysis, photocatalysis, adsorption, separation, and sensing to biomedicine. *Chem Soc Rev* 2016; **45**: 3479–563.
33. Yang XY, Chen LH, Li Y *et al.* Hierarchically porous materials: synthesis strategies and structure design. *Chem Soc Rev* 2017; **46**: 481–558.
34. Li Y, Fu ZY, Su BL. Hierarchically structured porous materials for energy conversion and storage. *Adv Funct Mater* 2012; **22**: 4634–67.
35. Chen LH, Li Y, Su BL. Hierarchy in materials for maximized efficiency. *Natl Sci Rev* 2020; **7**: 1626–30.
36. Blin JL, Léonard A, Yuan ZY *et al.* Hierarchically mesoporous/macroporous metal oxides templated from polyethylene oxide surfactant assemblies. *Angew Chem Int Ed* 2003; **42**: 2872–5.
37. Chen LH, Sun MH, Wang Z *et al.* Hierarchically structured zeolites: from design to application. *Chem Rev* 2020; **120**: 11194–294.
38. Ren TZ, Yuan ZY, Su BL. Thermally stable macroporous zirconium phosphates with supermicroporous walls: a self-formation phenomenon of hierarchy. *Chem Commun* 2004: 2730–1.
39. Su BL, Vantomme A, Surahy L *et al.* Hierarchical multimodal mesoporous carbon materials with parallel macrochannels. *Chem Mater* 2007; **19**: 3325–33.
40. Yuan ZY, Zhou W, Su BL. Hierarchical interlinked structure of titanium oxide nanofibers. *Chem Commun* 2002: 1202–3.
41. Chen LH, Li XY, Tian G *et al.* Highly stable and reusable multimodal zeolite TS-1 based catalysts with hierarchically interconnected three-level micro–meso–macroporous structure. *Angew Chem Int Ed* 2011; **50**: 11156–61.
42. Machoke AG, Beltrán AM, Inayat A *et al.* Micro/macroporous system: MFI-type zeolite crystals with embedded macropores. *Adv Mater* 2015; **27**: 1066–70.
43. Shen K, Zhang L, Chen X *et al.* Ordered macro-microporous metal-organic framework single crystals. *Science* 2018; **359**: 206–10.
44. Zhang Q, Mayoral A, Terasaki O *et al.* Amino acid-assisted construction of single-crystalline hierarchical nanozeolites via oriented-aggregation and intraparticle ripening. *J Am Chem Soc* 2019; **141**: 3772–6.
45. Sun MH, Zhou J, Hu ZY *et al.* Hierarchical zeolite single-crystal reactor for excellent catalytic efficiency. *Matter* 2020; **3**: 1226–45.

46. Sun MH, Chen LH, Yu S *et al.* Micron-sized zeolite beta single crystals featuring intracrystal interconnected ordered macro-meso-microporosity displaying superior catalytic performance. *Angew Chem Int Ed* 2020; **59**: 19582–91.
47. Hung CT, Duan L, Zhao T *et al.* Gradient hierarchically porous structure for rapid capillary-assisted catalysis. *J Am Chem Soc* 2022; **144**: 6091–9.
48. Pérez-Ramírez J, Verboekend D, Bonilla A *et al.* Zeolite catalysts with tunable hierarchy factor by pore-growth moderators. *Adv Funct Mater* 2009; **19**: 3972–9.
49. Murray CD. The physiological principle of minimum work. *Proc Natl Acad Sci* 1926; **12**: 207–14.
50. Sun MH, Gao SS, Hu ZY *et al.* Boosting molecular diffusion following the generalized Murray's Law by constructing hierarchical zeolites for maximized catalytic activity. *Natl Sci Rev* 2022; **9**: nwac236.
51. Zhou B, Cheng Q, Chen Z *et al.* Universal Murray's law for optimised fluid transport in synthetic structures. *Nat Commun* 2024; **15**: 3652.
52. Bereciartua PJ, Cantín Á, Corma A *et al.* Control of zeolite framework flexibility and pore topology for separation of ethane and ethylene. *Science* 2017; **358**: 1068–71.
53. Xiong H, Liu Z, Chen X *et al.* In situ imaging of the sorption-induced subcell topological flexibility of a rigid zeolite framework. *Science* 2022; **376**: 491–6.
54. Hong H, Liu J, Huang H *et al.* Ordered macro–microporous metal–organic framework single crystals and their derivatives for rechargeable aluminum-ion batteries. *J Am Chem Soc* 2019; **141**: 14764–71.
55. Derouane EG, Gabelica Z. A novel effect of shape selectivity: molecular traffic control in zeolite ZSM-5. *J Catal* 1980; **65**: 486–9.
56. Derouane EG, André JM, Lucas AA. A simple van der waals model for molecule-curved surface interactions in molecular-sized microporous solids. *Chem Phys Lett* 1987; **137**: 336–40.
57. Weisz PB. Molecular shape selective catalysis. *Pure Appl Chem* 1980; **52**: 2091–103.
58. Docquir F, Norberg V, Toufar H *et al.* Infrared study on the adsorption behavior of methylamine in a series of large pore cationic zeolites: a further confirmation of three types of interaction between methylamine and zeolites. *Langmuir* 2002; **18**: 5963–6.
59. Su BL, Docquir F. Competitive adsorption of benzene and ammonia on NaEMT zeolite: a quantitative infrared study. *LANGMUIR* 2001; **17**: 3341–7.
60. Su BL, Norberg V, Hansenne C *et al.* Toward a better understanding on the adsorption behavior of aromatics in 12R window zeolites. *Adsorpt Int Adsorpt Soc* 2000; **6**: 61–71.
61. Su BL, Norberg V, Hansenne C. Infrared spectroscopic study on the location of benzene in KL zeolite upon coadsorption of ammonia and methylamine. *Langmuir* 2000; **16**: 1132–40.
62. Su BL, Norberg V. Location of benzene in NaBeta zeolite upon coadsorption of ammonia and methylamine: a further confirmation of molecular recognition effect in benzene adsorption in 12R window zeolites. *Langmuir* 2000; **16**: 6020–8.
63. de Mallmann A, Barthomeuf D. Change in location of benzene in faujasite upon coadsorption of NH_3 or HCl. *J Chem Soc Chem Commun* 1989: 129–30.
64. Fang W, Wang C, Liu Z *et al.* Physical mixing of a catalyst and a hydrophobic polymer promotes CO hydrogenation through dehydration. *Science* 2022; **377**: 406–10.
65. Wei Y, Parmentier TE, de Jong KP *et al.* Tailoring and visualizing the pore architecture of hierarchical zeolites. *Chem Soc Rev* 2015; **44**: 7234–61.
